# Experimental Evidence for the Interplay of Exogenous and Endogenous Factors on the Movement Ecology of a Migrating Songbird

**DOI:** 10.1371/journal.pone.0041818

**Published:** 2012-07-23

**Authors:** Emily B. Cohen, Frank R. Moore, Richard A. Fischer

**Affiliations:** 1 Department of Biological Sciences, The University of Southern Mississippi, Hattiesburg, Mississippi, United States of America; 2 Environmental Laboratory, United States Army Engineer Research and Development Center, Vicksburg, Mississippi, United States of America; University of Western Ontario, Canada

## Abstract

Movement patterns during songbird migration remain poorly understood despite their expected fitness consequences in terms of survival, energetic condition and timing of migration that will carry over to subsequent phases of the annual cycle. We took an experimental approach to test hypotheses regarding the influence of habitat, energetic condition, time of season and sex on the hour-by-hour, local movement decisions of a songbird during spring stopover. To simulate arrival of nocturnal migrants at unfamiliar stopover sites, we translocated and continuously tracked migratory red-eyed vireos (*Vireo olivaceus*) throughout spring stopover with and without energetic reserves that were released in two replicates of three forested habitat types. Migrants moved the most upon release, during which time they selected habitat characterized by greater food abundance and higher foraging attack rates. Presumably under pressure to replenish fuel stores necessary to continue migration in a timely fashion, migrants released in poorer energetic condition moved faster and further than migrants in better condition and the same pattern was true for migrants released late in spring relative to those released earlier. However, a migrant's energetic condition had less influence on their behavior when they were in poor quality habitat. Movement did not differ between sexes. Our study illustrates the importance of quickly finding suitable habitat at each stopover site, especially for energetically constrained migrants later in the season. If an initial period prior to foraging were necessary at each stop along a migrant's journey, non-foraging periods would cumulatively result in a significant energetic and time cost to migration. However, we suggest behavior during stopover is not solely a function of underlying resource distributions but is a complex response to a combination of endogenous and exogenous factors.

## Introduction

The movement ecology of organisms is influenced by processes operating across broad spatial and temporal scales and plays a primary role in determining the fate of individuals as well as the dynamics of the populations that they comprise [1]. Therefore, an examination of the causes and consequences of individual movement is fundamental to understanding the complexities of ecological systems. Movement tracks can be considered as a series of “phases” that are a function of the organisms' internal motivational state, intrinsic motion and navigational capabilities, and the environment through which it is moving [1]. Long-distance migration is an extreme example of a movement phase encompassing hundreds to thousands of kilometers and comprising up to a third of a songbird's annual cycle. During migration, songbirds have the distinct goal of arriving safely at a specific seasonal destination on time and in sufficiently good condition to secure local resources and/or enhance annual reproductive success (e.g., [2], [3]). Successful migration requires frequent “stopover” periods between flights. Stopover periods cumulatively far exceed the time spent in flight and largely determine the duration of the migratory period [4]. Given the time and energetic constraints of migration (e.g., [5]), movement decisions during stopovers are likely to have fitness consequences for individuals in terms of survival as well as time of season and energetic condition upon arrival at wintering and breeding sites [6].

Long-distance migrants arrive at unfamiliar stopover sites after long flights and must balance the need to access food resources while limiting energy expenditure and exposure to predation risk from avian predators attracted to movement [7], [8] with limited information about availability of habitat, locations of predators or distribution of food resources. Therefore, selection should act on migratory behavior during stopover to maximize refueling rates while minimizing time, energy expenditure and exposure to predation risk [9]. Migrant behavior during stopover periods are known to be influenced by exogenous factors including distribution of food (e.g., [10]), presence of predators [11] and density of potential competitors [12] and endogenous factors including a migrant's energetic condition (e.g., [13], [14]), time program [15] and sex [16]. Yet, the primary environmental factors influencing migrants during stopover periods, how migrants react behaviorally to these factors and how these reactions are influenced by motivational state, remain poorly understood [15]. Therefore, the objective of this study was to assess the effects and relative influence of exogenous and endogenous factors on the movement pattern of a songbird species during stopover. Our experimental design controlled for exogenous and endogenous factors to experimentally assess for the first time, as far as we are aware, the effects and relative influence of multiple factors on the hour-by-hour movement behavior of a migratory songbird during spring stopover. We translocated and released male and female migrants with and without energetic reserves into two replicates of three forested habitat types throughout spring migration. We continuously followed their movements throughout stopover while simultaneously characterizing the environment through which they were moving. We hypothesized that migrants would make movement decisions according to (1) habitat type, as characterized by abundance of food resources, (2) energetic condition, (3) time of season and (4) sex.

**Figure 1:**
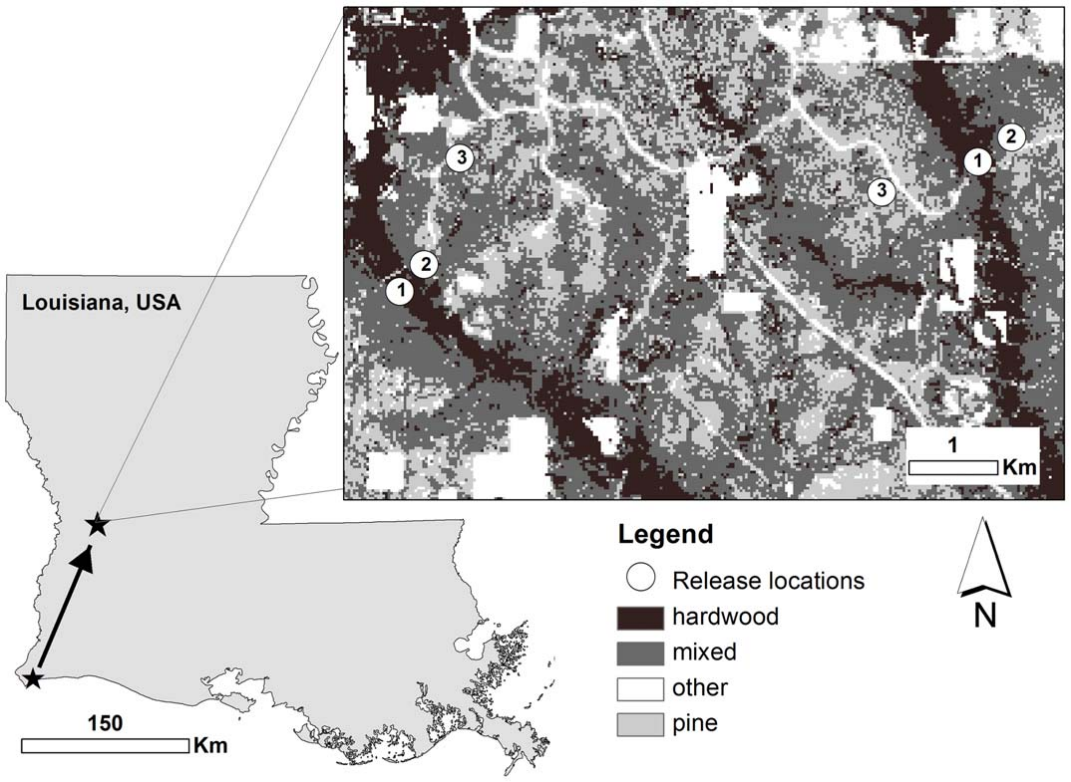
Map of study area. Migratory red-eyed vireos were captured at Johnson's Bayou during spring migration and translocated to Kisatchie National Forest Louisiana, USA. Inset map is of Kisatchie National Forest showing release locations in two replicates of three forested habitat types, hardwood (1) mixed (2), and pine (3).

**Table 1:** Summary of migratory red-eyed vireos released and tracked in two replicates of three forested habitat types.

	Number of individuals						
	Hardwood		Mixed		Pine		Total
Release habitat type	17		16		17		50
Energetic condition (no fat, with some fat)^a^	7	10	5	11	7	10	19, 31
Time of spring (first half, second half)^b^	10	7	8	8	8	9	26, 24
Sex (male, female)^c^	5	2	2	2	2	4	9, 8
Landscape (Drake, Bundick)	8	9	8	8	8	9	24, 26
Year (2007, 2008)	6	11	5	11	6	11	17, 33

a Migrants with no fat are less than lean body mass and migrants with fat are greater than lean body mass, see Materials and Methods.

b Median date 23 April (range, 4 April to 13 May).

c As estimates by wing chord (male ≥82 mm and female ≤76 mm wing chord, see [41]).

**Figure 2:**
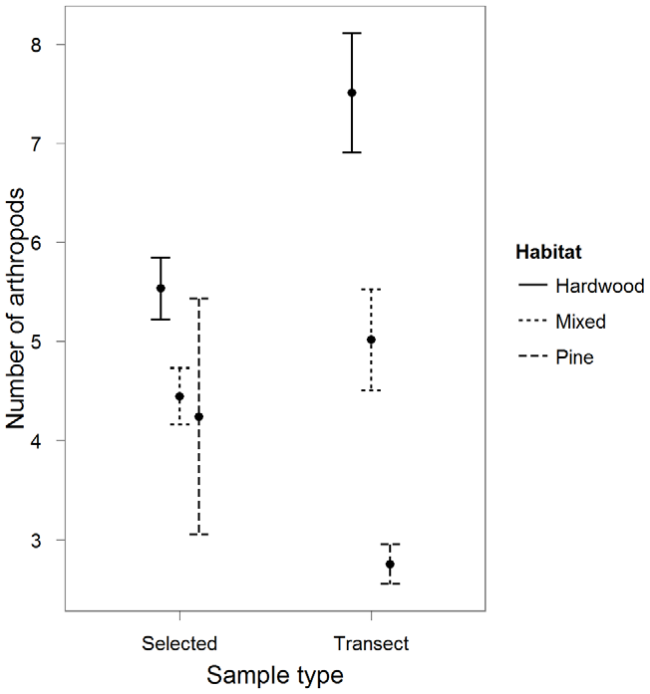
Mean number of arthropods (SE) in relation to forested habitat types and type of sample (location selected by migrants or systematically sampled on transect). The abundance of arthropods differed between habitat types (all *P*<0.001 from a zero-inflated hurdle model [see text]) and samples collected at systematically sampled locations (transects) had fewer arthropods than areas selected by migrants (selected; *P*<0.001) but there was a significant interaction between habitat type and type of sample (*P*<0.001). Within pine habitat (*n* = 37 selected, *n* = 133 transects) there were more arthropods in areas selected by migrants. The opposite was true within hardwood habitat (*n* = 213 selected, *n* = 132 transects) and there was no difference in arthropod abundance between sample types within mixed habitat (*n* = 150 selected, *n* = 130 transects).

Largely correlative evidence suggests that habitat selection occurs during migration, there are species specific patters of habitat associations between years [17] and multiple studies have demonstrated the distribution of migrants deviates from that expected based on availability of habitat types [7], 17,18,19. Some selection likely occurs prior to landing [20], [21] but passeriform night vision may only be sufficient for rough distinctions at night [22]. Further, distributions of migrants captured in multiple habitat types differed from the morning to later in the day [17], [23] and migrants radio-tracked at their capture locations moved further the first day of tracking [24], [25], [26]. All of this suggests that some level of habitat selection, possibly depending on the level of light the previous night, occurs during the morning of the day after landing. Therefore, we predicted that migrants would move the furthest and fastest the first morning at a stopover site during which time they would select among available habitat types. However, searching also corresponds to lost foraging time and a migrant will likely offset risk and energy expenditure by foraging within as restricted an area as contains the necessary food resources (e.g. [27], [9]). Thus, we predicted that migrants in forested habitat with abundant food would exhibit more area restricted movement and would select areas within those habitat types where food availability was greater. While the distribution of food is likely to be a primary factor influencing habitat selection during stopover, other attributes of habitat including vegetation cover to shield from predators [11], [28] and the density of competitors [12] are also likely to play a role in how habitat is used. We had *a priori* predictions about differential food abundance between forested habitat types in our study areas. Therefore, our predictions were related to food resources. However, we also quantified differences in vegetation structure and the distribution of potential competitors (transient migrants) and avian predators between habitat types.

**Table 2:** Abundance and distribution of branch sampled arthropods and transient migrants.

	Count model			Binomial model		
Effect	Estimate	Z	*P*	Estimate	Z	*P*
Arthropods (*n* = 795 samples, df = 21)						
Intercept	0.45±0.27	–	–	3.01±0.74	–	–
Mixed habitat a	1.53±0.32	4.83	<0.0001	−1.94±0.89	−2.18	0.03
Hardwood habitat a	2.03±0.31	6.66	<0.0001	−0.31±1	−0.31	0.76
Selected b	0.64±0.19	3.23	0.001	−0.62±0.47	−1.32	0.19
Date	0.03±0.03	0.76	0.45	−0.09±0.14	−0.69	0.49
Landscape	−0.1±0.07	−1.40	0.16	−0.10±0.25	−0.40	0.69
Year	−0.25±0.09	−2.97	0.003	−0.26±0.29	−0.87	0.38
Hour	0.005±0.01	0.40	0.69	−0.05±0.05	−1.06	0.29
Mixed habitat × selected c	−0.8±0.23	−3.52	<0.001	1.88±0.64	2.92	0.004
Hardwood habitat × selected c	−0.92±0.22	−4.19	<0.0001	1.14±0.67	1.71	0.09
Transient migrants (*n* = 99 days, df = 10)						
Intercept	−1.30±0.46	–	–	−1.56±0.65	–	–
Mixed habitat a	0.50±0.44	1.13	0.26	2.12±0.82	2.58	0.01
Hardwood habitat a	1.23±0.40	3.11	0.002	4.34±1.23	3.53	<0.001
Date	0.29±0.13	2.31	0.02	0.38±0.38	1.01	0.31
Landscape	0.89±0.31	2.86	0.004	4.55±1.19	3.82	<0.001

a relative to pine habitat.

b relative to transect samples.

c relative to pine habitat and transect samples.

The abundance and distribution of arthropods and transient migrants from Zero-inflated two-part models for count data (binomial model represents presence or absence and count model tests for relationships within positive counts). We also tested our expectation that arthropods would be greater in areas selected by migrants (selected) versus systematically sampled (transect) and that this relationship may differ with habitat type (interaction terms). The landscape of the habitat replicate and the year (2007 or 2008; transient migrants were only sampled in 2008) were not expected to differ but included as covariates.

**Figure 3:**
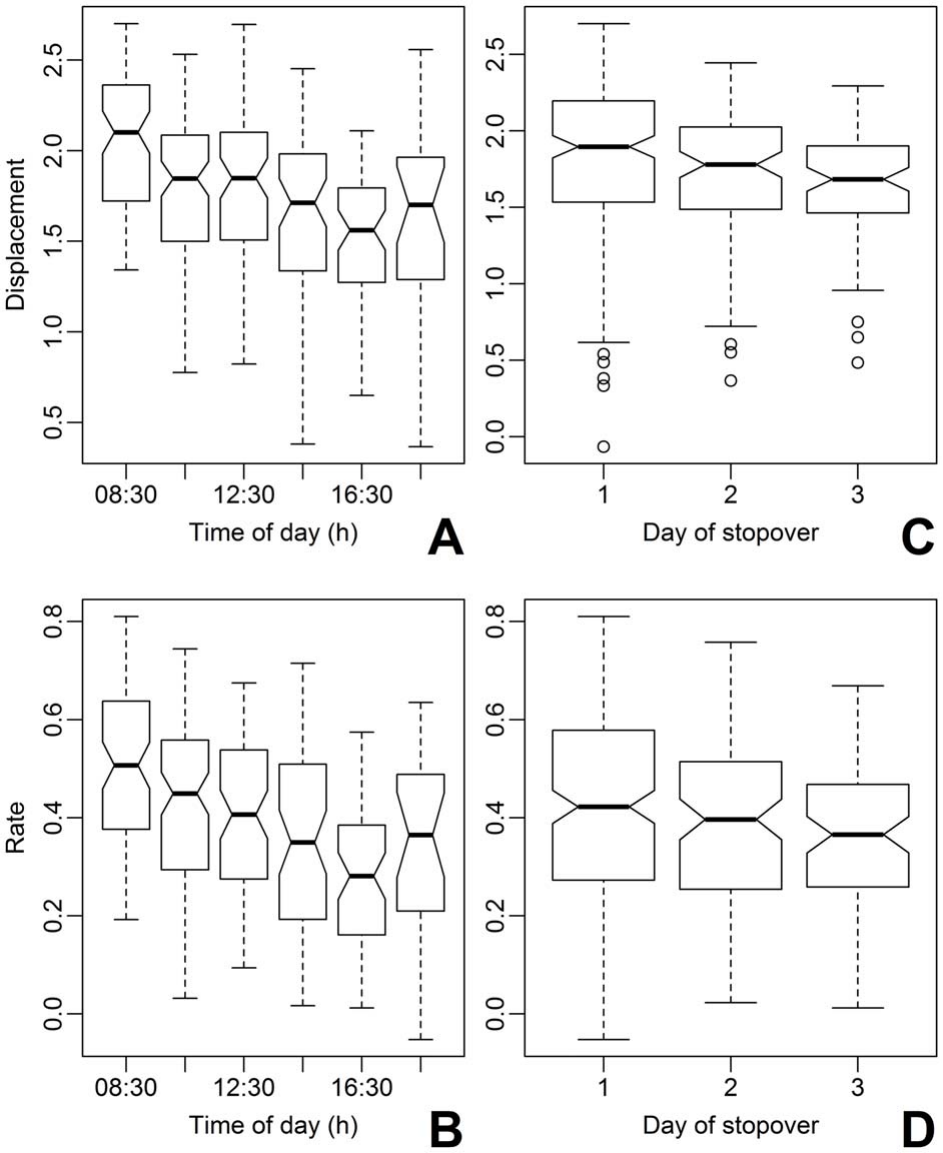
Model fitted movement rate and displacement distance of migratory red-eyed vireos in relation to hour of the day and day of stopover. Fitted values were extracted from a mixed model with seven fixed effects and the individual bird as a random component. Response variables were the log transformed linear displacement distance (m, A and C) and movement rate (m min^−1^, B and D) during two hour increments throughout the first three days of stopover. Box plot whiskers depict the minimum and maximum values and around the median, indicated by the bold line. Medians differ between factors when notched areas do not overlap.

We also made predictions regarding the influence of endogenous factors; condition, time program and sex on movement. Energetically constrained individuals are under more pressure to replenish fuel stores [29] and forage more rapidly and over a greater area to access prey resources [30], [13]. Therefore, we predicted that migrants arriving at stopover sites with little or no energetic reserves would move faster and further, to find and acquire available food resources, than migrants arriving with greater fuel stores. We expected this to be particularly true in forested habitat with reduced food resources where migrants with reduced energetic reserves are under the most pressure to locate food resources. The constraint to minimize time spent on migration [5] implies that migrants arriving later in the season are under more pressure to replenish fuel stores quickly [15]. Therefore, we predicted that migrants arriving later in the season would move further and faster relative to individuals arriving earlier in the season. We consider time of season an endogenous factor in that it is likely to reflect an individual's time program [15]. In some species, males migrate earlier (e.g. [31]), gain mass faster during stopover [32] and arrive at breeding sites earlier than females [33]. Consequently, males may be motivated to forage faster during stopover in order to migrate faster [34]. As a result, we predicted that males would move further and faster than females.

**Table 3:** Influence of endogenous and exogenous factors on migrant movement (linear displacement and movement rate).

Effect	Estimate ± SE	*F*_df_	*P*
Linear displacement (log transformed)			
Intercept	2.43±0.24	–	–
Condition	−0.08±0.08	29.07 _1,46_	<0.0001
Date	0.17±0.06	25.24 _1,320_	<0.0001
Habitat a	−0.02±0.09	0.26 _2,320_	0.77
Hour	−0.07±0.03	34.86 _1,320_	<0.0001
Day	−0.18±0.04	12.70 _1,320_	<0.001
Landscape	0.28±0.11	6.90 _1,46_	0.01
Year	−0.13±0.11	0.98 _1,46_	0.33
Condition × Habitat	−0.05±0.03	2.88 _2,320_	0.06
Condition × Date	0.01±0.03	0.04 _1,320_	0.84
Condition × Hour	−0.01±0.01	0.72 _1,320_	0.40
Condition × Day	0.06±0.02	6.72 _1,320_	0.01
Habitat × Hour	0.01±0.01	1.48 _2,320_	0.23
Rate (log transformed)			
Intercept	0.66±0.08	–	–
Condition	−0.06±0.03	24.90 _1,46_	<0.001
Date	0.07±0.02	22.14 _1,320_	<0.001
Habitat a	−0.01±0.03	1.01 _2,320_	0.37
Hour	−0.03±0.01	48.99 _1,320_	<0.001
Day	−0.05±0.02	9.42 _1,320_	0.002
Landscape	0.15±0.04	16.94 _1,46_	<0.001
Year	−0.10±0.04	5.49 _1,46_	0.02
Condition × Habitat	−0.01±0.01	1.04 _2,320_	0.35
Condition × Date	−0.01±0.01	0.44 _1,320_	0.51
Condition × Hour	0.0002±0.002	0.04 _1,320_	0.84
Condition × Day	0.02±0.01	3.48 _1,320_	0.06
Habitat × Hour	0.003±0.004	0.57_2,320_	0.57

a From pine to mixed to hardwood.

Models include the individual migrant as a random factor (50 individuals) to account for the nonindependence of movement from the same individuals between time periods (382 two-hour movements); SE, standard error.

**Table 4:** Influence of factors on migrant habitat selection.

Effect	Estimate ± SE	*F*_df_	*P*
Intercept	1.03±0.30	–	–
Release habitat type	1.04±0.30	10.47 _1,45_	<0.001
Day	−0.08±0.04	1.15 _1,328_	0.06
Hour	0.08±0.03	3.46 _1,328_	0.28
Landscape	0.12±0.16	0.43 _1,45_	0.52
Year	−0.19±0.17	1.33 _1,45_	0.26
Release habitat × Hour	−0.03±0.01	3.59 _2,328_	0.03

We used mixed-effects models with the individual bird as the random component to control for the non-independence of habitat use for the same individual between time periods (06∶30 to 18∶30 h) and days (1 to 3). Models include fixed effect for the influence of the release habitat type (Pine, Mixed, or Hardwood) and day of stopover (1 to 3) on habitat use at the end of each two-hour period. We also expected the release habitat to decline with increasing time of day (Release habitat type x Hour) and included the landscape and year (2007 or 2008).

## Materials and Methods

### Ethics statement

This experiment was carried out in accordance with the Ornithological Council's guidelines to the use of wild birds in research and was approved by The University of Southern Mississippi's institutional animal care and use committee (protocol #1092210). Other permits were from the United States Department of the Interior bird banding laboratory (permit #21221) and Fish and Wildlife Service (permit #MB75836-3) and the Louisiana Department of Wildlife and Fisheries (permit #LNHP-11-058).

### Study Species

The focal species of this study was the red-eyed vireo (*Vireo olivaceus*), a Neotropical-Nearctic migratory songbird common throughout eastern deciduous forest of North America. The species is primarily a canopy foliage-gleaner but uses diverse substrates while foraging on a variety of arthropod taxa during migration [35]. Red-eyed vireos are most often found in hardwood habitat during breeding [35] but are known to occupy both bottomland hardwood and pine habitat with hardwood understorey during spring migration [36].

**Figure 4:**
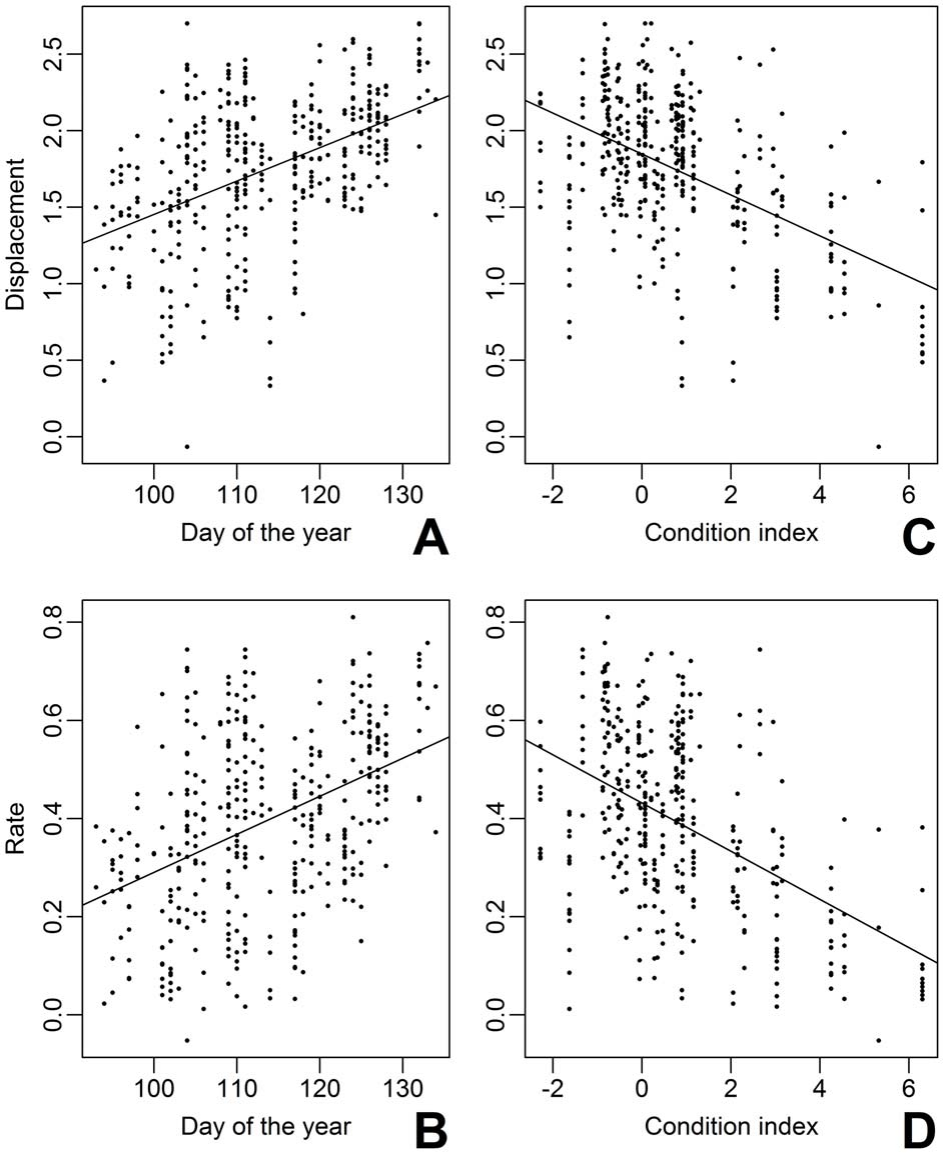
Model fitted movement rate and displacement distance of migratory red-eyed vireos in relation to time of spring and energetic condition. Fitted values were extracted from a mixed model with seven fixed effects and the individual bird as a random component on the log transformed linear displacement distance (m, A and C) and movement rate (m min ^-1^, B and D) during two hour increments throughout the first three days of stopover. Migrants were released and tracked from early to late spring (4 April to 13 May, 2007 and 2008) in a range of energetic conditions (index is relative to lean body mass [0, see text].

**Figure 5:**
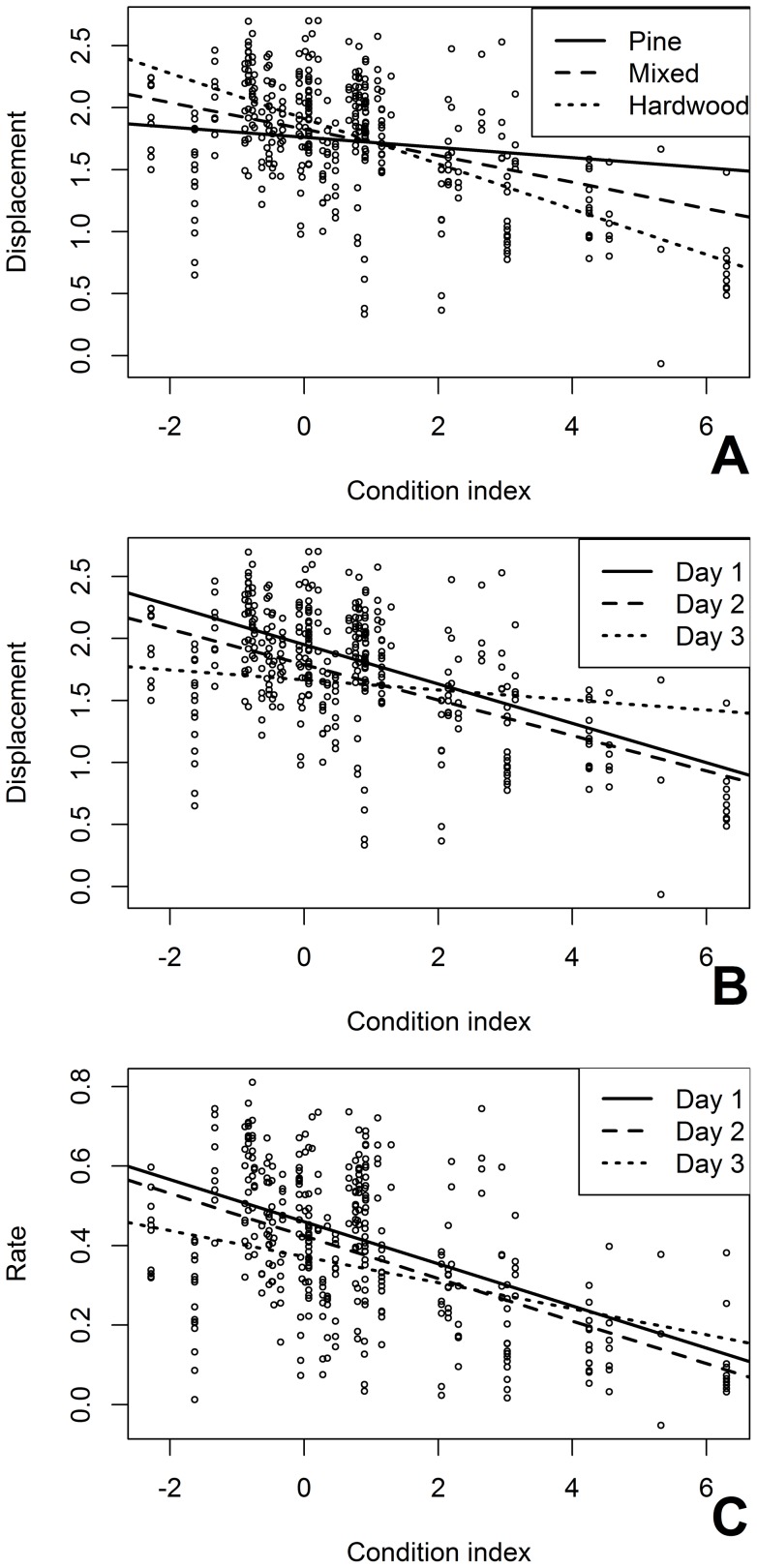
The influence of energetic condition on migrant movement varies with habitat type and day of stopover. Fitted values were extracted from mixed models with seven fixed effects and the individual bird as a random component on the log transformed linear displacement distance (m) and movement rate (m min^−1^) during two hour increments throughout the first three days of stopover. Model fitted linear displacement distance of migratory red-eyed vireos in relation to energetic condition (condition index is relative to lean body mass [0, see text]) in three forested habitat types and on the first through third day of stopover (A and B). Model fitted movement rate in relation to energetic condition on the first, second and third day of stopover (C).

### Translocation and tracking

Red-eyed vireos were captured in southwestern Louisiana near the Gulf of Mexico at Johnson's Bayou (29° 45′ N 93° 30′ W, Figure 1). Red-eyed vireos do not breed at Johnson's Bayou, so individuals captured there were known to be transient. The netting effort covered the available forested habitat and nets were kept open most of the day so we could also be relatively certain that the migrants we captured had arrived either the capture day or the previous afternoon. Upon capture, migrants were banded with a US Fish and Wildlife Service band and a unique combination of colored leg bands. Morphometric measurements were taken and subcutaneous fat was assessed [37].

The transient red-eyed vireos were transported the afternoon or evening of the day of capture approximately 143 km to the Vernon Unit of the Calcasieu Ranger District in Kisatchie National Forest, Louisiana (30° 57′ N 93° 08′ W, Figure 1). This site was chosen for several reasons. First, it has a variety of forested habitat types representative of those found throughout the East Gulf Coastal Plain [38]. Cover types in and around Kisatchie National Forest include upland longleaf pine savannas, bottomland hardwood, mixed pine and hardwood, planted pine, and harvested or open areas. Second, a high density of spring migrants use this area as a stopover site, based on weather radar observations [39]. Third, it is an inland site so habitat selection would not be constrained by adjacency to an ecological barrier. Red-eyed vireos also commonly breed at this site so it would not have been possible to accurately establish the status (breeding or migratory) of migrants captured there. Migrants were held in individual cages for 13.5 to 22 hours (17.43±2.49 hours) and provided with food and water *ad libitum*. Red-eyed vireos were known to acclimate well to captivity [34] and lost little mass during the night we held them (1.12±0.61 g for the period of captivity, *n* = 50 birds with mass 16.76±1.80 g). The evening of the capture day migrants were fitted with radio-transmitters weighing less than 3.5% of mean lean body mass (model LB-2, 0.52 g, Holohil Systems Ltd. Ontario, Canada).

Red-eyed vireos with and without fat reserves were released and tracked continuously in three forested habitat types nested in two landscapes approximately 3 km apart (Figure 1) from early April through mid-May of each year (Table 1). We chose release sites in the most abundant forested habitat types also characteristic of the East Gulf Coastal Plain region and thought to differ in available food resources: upland pine savanna (pine), bottomland deciduous forests along creeks (hardwood), and an intermediate between the two (mixed). Hardwood release sites were in bottomland hardwood floodplains surrounding creeks and pine and mixed release sites were in locations near each hardwood release site but predominately surrounded by pine and mixed habitat, respectively (Figure 1). The area surrounding each set of release sites are referred to as Drake and Bundick landscapes, using the names of the creeks that pass through them.

An energetic condition index was calculated for each individual to reflect the proportion of body mass attributed to fat (see, [40]). Size specific fat-free masses were calculated from regression curves of wing chord length on mass for red-eyed vireos captured without fat (fat score = 0) during spring at Johnson's Bayou (1998 to 2006, *n* = 1775). The mass of each translocated bird was recorded in the morning just prior to release with an electronic scale to the nearest 0.1 g. The size specific fat-free mass for that individual (as determined by their wing chord) was subtracted from the release mass to calculate the index of release condition. Therefore, the condition index reflects the amount of mass attributed to fat that an individual migrant carries. It does not have units but is relative to the fat free body mass (condition index  = 0). Increasingly positive condition indices indicate greater fat reserves and decreasing negative condition indices indicate no fat reserves and decreasing mass. While no single index of body condition is without some error [41], during migration fat levels are highly variable and our index was also correlated with fat score (Pearson's product-moment correlation t_49_ = 7.70, *r* = 0.74, *P*<0.0001). The ordinal day of the release date was used for the time of season. Sex was determined for 17 of the 50 migrants (male ≥82 mm and female ≤76 mm wing chord, [42]).

Migrants were continuously radio-tracked with locations taken every 15 min for up to three days of stopover following release. To minimize observer impacts, migrants were located to within 30 to 50 m and then their locations were circled to verify the accuracy of the location. In 2007, we tracked continuously for the first five and last hour of each day and in 2008 we tracked continuously from dawn to dusk. The predominant habitat type in the 30 m surrounding each individual location was recorded. When it was not possible to observe a tagged migrant we recorded the location as either active (with some signal fluctuation) or stationary (with no signal fluctuation for ≥30 s), we calibrated this technique and found it to be accurate when conditions were not windy (and not recorded). When it was possible to visually observe a tagged migrant, we recorded continuous observations of foraging success rate as the number of successful attacks per second.

### Characterization of exogenous factors

We characterized the vegetation structure and composition and the abundance and distribution of arthropods and transient migrants within each forested habitat type by sampling along six 1200 m transects passing through each release site and remaining within the habitat type. Vegetation structure and composition were quantified from six sampling plots [43] evenly spaced along each transect. Circular plots (11.3 m radius) were established within which we identified each tree that was greater than or equal to 5 cm in diameter at breast height (1.4 m) to species and recorded the diameter. We calculated the total basal area of the 15 most common tree species and summarized the vegetation structure with eight variables: total basal area, number of shrubs, shrub layer cover (1.5 to 2.5 m above the ground), canopy cover, canopy height, herbaceous layer height, percentage of live ground cover and percentage of dead leaf litter that is pine needles.

We tested our expectation that food abundance (number of arthropods per sample) varied with forested habitat type (hardwood > mixed > pine) by collecting arthropod samples every 100 m on each transect three times per spring: early (13 to 18 April), middle (26 April to 2 May), and late (7 to 12 May) using canopy branch clipping, a method that has been shown to be effective in measuring arthropod prey density on and near vegetation used by foliage-gleaning birds [44]. We alternated the times of day arthropod samples were collected in each habitat type. Transect arthropod samples were collected at the same transect locations each time of season and year, but were not collected from the same branches or necessarily from the same trees at those locations. Arthropod samples were collected using a cotton hoop net on an extending survey pole to encompass the end of a branch 4 to 6 m above the ground [44]. All collected arthropods were identified to order and length measured. We choose branches approximately 0.25 m long from the closest pine (*Pinus* spp.), oak (*Quercus* spp.), or sweetgum (*Liquidambar styraciflua*) tree that was greater than 7 m tall and had a branch 4 to 6 m above the ground.

We also tested for differences in food abundance from early to late spring. We characterized the number of transient migrants and avian predators associated with forested habitat types from daily avian surveys (conducted in 2008) along 500 m sections of the six transects. One person conducted all surveys, walking at a constant rate and recording all avian species seen or heard within 50 m of transects. The surveyor alternated daily between landscapes, sampling three transects in one of the two landscapes and systematically rotating the order of habitat types surveyed (16 sets of surveys at Drake and 17 at Bundick).

We tested our prediction that migrants select areas within forested habitat types where there is greater food abundance by additionally sampling for arthropods at locations selected by migrants. We used the same methodology described above to sample arthropods at locations along migrants' movement paths. For the majority of migrants tracked in 2008, we returned within ten days to take arthropod samples at the first location of every second hour of locations from the first day of stopover. We tested for differences in the number of arthropods per sample between these samples and the samples collected systematically on the transects in each habitat type.

### Analyses

A permutational multivariate analysis of variance was used (adonis function in library vegan for R) to quantify vegetation structure and tree community composition between habitat types and the two landscapes. We tested our expectation that habitat types differed in food abundance (hardwood > mixed > pine) and our prediction that migrants select areas within habitat types with greater food availability (selected > transect) with a zero-inflated negative-binomial model for count data (hurdle function in library pscl for R). Repeated measures tests were not necessary because while samples were collected at the same locations along transects, they were not collected from the same branches or necessarily from the same trees at those locations and branches were removed for sampling. In addition to habitat type and type of sample (transect or selected), we also included day of season (date) and hour of the day (hour) to account for any temporal differences in food abundance. We also tested for an interaction between habitat and type of sample (transect vs. selected sample). We used maximum likelihood and AIC to determine the appropriate distribution (negative-binomial) and used a two part model to account for the high number of zeroes [45]. Two-component models first model the presence of arthropods using a generalized linear model with a logit link and binomial error and then model the abundance of arthropods, where they occurred, with a second generalized linear model with a negative-binomial distribution and a log link [45]. We present results from both models but focus primarily on abundance.

For species that migrate through the region but also breed there, we conservatively estimated the number of transient migrants. We used the minimum number of individuals of each species detected on a survey as an estimate of the number of breeding individuals of that species within the sampling area of that transect. We subtracted this species-transect specific estimate from the number detected each day on each transect daily on each transect. We tested for differences in the total number of transient migrants between habitat types, landscapes and times of season. We used maximum likelihood and AIC to determine the appropriate model distribution (Poisson) and again used a two-component model as described above to account for the high number of zeros in detections of transient migrants for arthropods (hurdle function in library pscl for R, [45], [46]).

Movement patterns during the first three days of stopover were quantified for each individual as the linear displacement (the log-transformed linear distance between the first and last location of the time period, m) and rate (the log-transformed cumulative distance between all locations divided by the time in a time period, m min^−1^) during two-hour increments (from 6∶30 to 18∶30 daily). We used linear mixed-effects models including the three fixed effects controlled for in our study design: energetic condition at release (condition), day of the year (date), and habitat type during each time period (habitat). Two additional fixed effects and one interaction were included to test our predictions regarding temporal variability of movement, hour of day (hour) and day of stopover (day) and hour x habitat. Finally, we included four interaction terms to test our predictions regarding the differential influence of energetic condition within habitat types and through time (condition x habitat, condition x hour, condition x day, condition x date). The individual migrant was included as the random component to allow for correlations of observations from the same individual between time increments which allows intercepts and slopes to vary between individuals when testing for significant mean slopes of fixed effects (REML function in library nlme for R, [45], [46], [47]). It was possible to estimate sex for 17 individuals: nine males and eight females, so we fit the same models (of all fixed and random effects) for the subset of individuals to test for differences between sexes.

We tested our predictions regarding the direction and timing of habitat selection using a linear mixed-effect model with the individual bird as the random component and the release habitat type (release type), the day of stopover (day), and an interaction between the release habitat type and the hour of the day (release type x hour) as explanatory variables of the habitat type at the end of each time period. We used foraging observations to quantify differences in foraging success between habitat types. We quantified prey capture rates (prey sec^−1^) whenever it was possible to visually identify and observe a tagged migrant and used an ANOVA to test for differences between habitat types. Maximum likelihood and Akaike Information Criteria (AIC) were used to determine the appropriate random effects structure for the movement pattern and habitat selection models (random intercept and random slope) and restricted maximum likelihood to test fixed effects [45]. All analyses included the landscape within which the release site or transect was nested (Drake or Bundick) and the year (2007 or 2008, excluding transient migrants which were only surveyed in 2008) as covariates. Finally, we assessed the fit of models by checking the residuals of each explanatory variable for normality and homoscedasticity by plotting the residuals against predicted values and found that model assumptions were not violated. Tests are 2-tailed with a significance level set to alpha  = 0.05. Analyses were conducted in R 2.12.2 [47]. Means ± SD are reported unless stated otherwise.

## Results

### Characterization of exogenous factors

The vegetation structure differed by habitat type (*pseudo-F*_2, 35_ = 26.02, *P*<0.001) but not by landscape (*pseudo-F*_1, 35_ = −0.33, *P* = 0.999) as did the tree community (habitat type *pseudo-F*_2, 35_ = 15.831, *P*<0.001; landscape *pseudo-F*_1, 35_ = 1.367, *P* = 0.244). The three release habitat types did not differ in canopy height (pine 25.58±4.01 m, mixed 22±8.17, hardwood 27.25±6.84), herbaceous layer height (pine 0.47±0.20 m, mixed 0.28±0.24, hardwood 0.31±0.09) or total basal area (pine 0.91±0.26 m^2^, mixed 0.74±0.37, hardwood 1.27±0.37) but pine habitat had less shrub layer cover (pine 17.5±20.06%, mixed 45.33±23.40, hardwood 28.67±20.39), more live ground cover (pine 43.00±13.37%, mixed 20.5±15.34, hardwood 20.83±14.30), and more leaf litter as pine needles (pine 97.92±4.48%, mixed 50.00±17.91, hardwood 8.08±10.11), mixed habitat had more shrubs (pine 17.58±22.08, mixed 64.17±33.15, hardwood 9.83±11.22), and hardwood habitat had greater canopy cover (pine 89.03±7.88%, mixed 95.08±4.54, hardwood 99.52±0.68).

As expected, our arthropod sampling revealed differences between habitat types. Where present, arthropods were more abundant in hardwood and mixed than in pine habitat (Table 2). Arthropods were also more abundant in areas selected by migrants than in systematically sampled locations (Table 2). However, there was a significant interaction between the type of sample and the habitat type within which it was collected (Table 2). Within pine habitat, there were more arthropods in areas selected by migrants (4.24±7.23, *n* = 37) than on transects (2.75±2.29, *n* = 133), whereas in mixed and hardwood habitat, there were fewer arthropods in selected areas (mixed 4.44±3.47, *n* = 150; hardwood 5.54±4.55, *n* = 213) versus on transects (mixed 5.01±5.82, *n* = 130; hardwood 7.51±6.91, *n* = 132, Figure 2). In addition, there were no differences in arthropods between times of day, landscapes or times of season but there were fewer arthropods in 2008 than in 2007 (Table 2).

Surveys for transient migrants found more frequently in mixed and hardwood than in pine and, when encountered, were more abundant in hardwood than in pine (Table 2). Transient migrants were also encountered more often and more abundant in the Bundick than in the Drake landscape (Table 2). Finally, transient migrants were slightly more abundant earlier in the season (Table 2). There were more hawks detected on transects in the Bundick (*n* = 13) than in the Drake landscape (*n* = 5) but detections were in all three habitats (*n* = 12 hardwood, *n* = 2 in mixed, *n* = 4 in pine) on both sets of transects.

### Movement Ecology

Migrants were rarely stationary (14% of *n* = 2177 locations), moving up to 2,347 m during the first day of stopover (mean linear displacement 618±519 m, *n* = 50). Migrant movement was influenced by exogenous and endogenous factors and varied temporally during stopover (Table 3). The furthest and fastest movements occurred during the first two hours of the release day and then gradually decreased with the hour of the day and the day of stopover (Figure 3).

Migrants did not vary their movement distance or rate while in different habitat types throughout the day (Table 3). However, as predicted, migrants moved to select habitat the morning of release, gradually moving out of pine and into mixed and hardwood habitat. During the first two hours of the first day, migrants released in pine moved further (405±348 m) than those released in mixed (197±224 m) or in hardwood habitat (147±178 m). The release habitat type strongly influenced subsequent habitat use but was most influential during the first hours of the first day of stopover (release habitat x hour, Table 4). The first two hours after release, the majority of migrants remained in their release habitat type but by the afternoon, as well as on subsequent days of stopover, the majority of migrants released in all habitat types were in either mixed or hardwood habitat. There were no differences in habitat selection between years or landscapes (Table 4). Migrants released in the habitat replicates in the Bundick landscape also moved both faster and further than did those released in the Drake landscape (Bundick displacement 157±195 m and rate 2.44±2.19 m min^−1^ m for two hour periods; Drake displacement 116±167 m and rate 1.49±1.76 m min^−1^, Table 3).

Foraging observations did not support an initial stationary period for acclimation prior to flying or foraging. Habitat type influenced minimum latency to forage and to successfully catch prey in that migrants were observed foraging as early as one minute after release in hardwood, 22 min after release in mixed and 48 min after release in pine and successful foraging was observed as early as one minute after release in hardwood, 32 min after release in mixed and an hour after release in pine. Migrants caught prey more quickly in habitat types characterized by greater food resources (F _2, 26_ = 3.37, *P* = 0.04, *n* = 29 observations totaling 65 min, from 14 individuals). Capture rates in pine were significantly less than in hardwood (*P* = 0.04), but capture rates did not differ between mixed (0.04±0.02 prey s^−1^, *n* = 5) and pine (0.01±0.003 prey s^−1^, *n* = 15, *P* = 0.41) or mixed and hardwood (0.05±0.02 prey s^−1^, *n* = 9, *P* = 0.72).

Our predictions regarding the influence of energetic condition and time of season on movement during stopover were supported. Migrants were released in a range of energetic conditions from well above (index  = 6.3) to well below (index  = −2.28) lean body mass (index  = 0). Migrants arriving in poorer energetic condition moved faster and further than migrants with greater fat reserves (Table 3, Figure 4). However, the influence of energetic condition also varied with habitat type and day of stopover (Table 3, Figure 5). Energetic condition strongly influenced how far migrants moved (leaner birds moved further) when they were in hardwood habitat and some influence when they were in mixed habitat but had little influence on how far migrants moved when they were in pine habitat (Figure 5). Arrival energetic condition also had little influence on movement by the third day of stopover (Figure 5). Additionally, migrants arriving later in the season moved both faster and further than individuals released earlier in the spring (Table 3, Figure 4). There were no differences in movement between sexes (linear displacement male 144.66±203.76 m, female 104.71±154.12 m, *F* = 2.40 _1,12_, *P* = 0.15; rate male 2.36±2.2 m min^−1^, female 1.35±1.77 m min^−1^, *F* = 2.14 _1,12_, *P* = 0.17; *n* = 17 birds, *n* = 132 observations).

## Discussion

Long-distance migrants arrive at stopover sites with vastly different energetic reserves and a short period of time to safely rest and/or replenish fuel reserves while in diverse and unfamiliar landscapes. Given that migrants arrive in these landscapes with little to no information about resources and differ in their time and/or energetic constraints, it is not surprising that movement during stopover is considerably variable. Nevertheless, our experimental design and continuous monitoring of movement enabled us to draw strong inferences about how red-eyed vireos respond to exogenous and endogenous factors during spring stopover.

### Influence of exogenous factors

We found support for our expectations that migrants move to explore habitat the morning after arrival and move further upon release in habitat types characterized by reduced food resources. The time until attempted and successful foraging was also habitat-dependent and negatively related to food abundance. There are energetic and time costs associated with a requisite initial period prior to foraging at stopover sites. If necessary at each stop along a migrant's journey, non-foraging periods would affect the optimal energy load and duration of stay [5], [48] cumulatively resulting in a significant energetic and/or time cost to migration [49]. Observed mass losses of migrants captured multiple times during stopover suggests an initial non-foraging period (reviewed in, [50]) but the effect of the capture handling time could not be eliminated in these banding studies [9]. We found no evidence that migrants acclimate to habitat prior to foraging; stationary behaviors were observed only slightly more often during the first two hours of the day and migrants began foraging almost immediately upon release in habitat with abundant food. Delingat et al. [9] also observed foraging from one minute to half an hour after moving and releasing Northern wheatears (*Oenanthe oenanthe*), presumably into habitat with abundant food.

The necessity of finding food implies that migrant movement would be primarily influenced by the distribution of food resources. This is supported by correlative evidence for habitat selection based on food abundance [18], [51], [52] and migrant distributions in relation to changes in food availability at different scales (reviewed in, [53], [20]). We found support for migrants selecting habitat characterized by greater food resources. Migrants released in hardwood largely stayed in hardwood whereas migrants released in mixed and pine moved into hardwood. This pattern was consistent in both landscapes and was moreover also true for migrants with greater fat reserves, presumably under less pressure to locate food resources [30], [29]. These results suggest that it is beneficial to a migrant to search for high quality habitat characterized by greater food even with the potential energetic costs and increased likelihood of exposure to avian predators attracted to movement. It is also possible that foraging opportunities may be functionally different between habitats. Red-eyed vireos may experience more difficulty finding and/or taking prey items within pine vegetation, though we did observe individuals foraging on pine, and we detected Lepidoptera larvae on pine needles, preferred food source of the species [35].

If movement of migratory songbirds during stopover is largely a function of the distribution of food resources, then we expected migrants to not only select habitat types with greater food resources but also to select areas within habitat types where food availability is greater. When migrants were in habitat characterized by less abundant food (pine), they selected locations within that habitat type with greater food abundance than at random locations. Conversely, once in habitat characterized by more abundant food (hardwood), red-eyed vireos selected locations with considerably less abundant food than expected by chance alone. Champlin et al. [54] also found migrant habitat use did not change with food abundance within hardwood habitat. This implies that migrants may search for areas with sufficient food as opposed to areas with the most abundant food supply. Further, migrants gradually moved through the landscape during the day and did not show any indication of defending territories once in high quality habitat.

Counter to our expectation, migrants did not move in a more restricted area within habitat with more abundant food. Interestingly, migrants also moved differently between the two landscapes within which habitat replicates were embedded. Migrants moved faster and further in the Bundick landscape than in the Drake landscape, yet the two did not differ in food abundance or in vegetation structure or composition. The Bundick landscape was characterized by more en route migrants and more avian predators, and we know that migratory birds are responsive to competition for resources (e.g., [12]) and predation pressure (e.g., [27], [55]) during stopover. Factors other than food abundance may influence movement decisions once settled in a habitat, especially within high quality habitat where food may not be limited.

### Influence of endogenous factors

Energetic condition upon release strongly influenced movement of red-eyed vireos during stopover. As predicted, migrants with reduced fat stores moved further and faster during stopover (see also, [13], [14]), consistent with pressure to replenish depleted fuel stores necessary to continue migration in a timely fashion [5]. Migrants with greater energetic reserves may move at a slower rate and over shorter distances to conserve fat stores and reduce risk of predation (see [29], [55]). At the within-habitat scale, energetically constrained red-eyed vireos and thrushes foraged at faster rates using more diverse substrates and maneuvers relative to individuals with greater fat stores [30], [29]. However, two other species tracked during stopover did not exhibit condition-dependent movement [16], [26], [56]. We found the influence of energetic condition on movement behavior was most pronounced when migrants were in high quality habitat. Therefore, a migrant arriving at a stopover site with poor quality habitat, where food resources are scarce, may be more influenced behaviorally by exogenous than endogenous factors. Our finding that arrival energetic condition influenced movement through the second day of stopover suggest that migrants may take several days to replenish fuel stores, even in landscapes with abundant high quality habitat.

Movements of migrants that are later in relation to their destination are expected to reflect higher fuel deposition rates during stopover [15]. Indeed, we found migrants moved faster and further as the season progressed, though it was not possible to determine the specific breeding destinations, hence the remaining distance, of the red-eyed vireos that we tracked. Wilson's warblers (*Wilsonia pusilla*) moved faster and further later in the spring, which may have been related to reduced availability of food resources late in the season [16]. However, we found no support for a seasonal change in abundance or distribution of food. Although our results support the expectation that movement varies in relation to an individual's time program, we could not eliminate the possibility that other factors influenced seasonal movement such as the abundance of transient migrants, which increased slightly later in the spring.

There was no difference in movement between male and female red-eyed vireos, though it was only possible to identify sex for a subset of individuals. In an effort to minimize our impact we chose not to take blood samples during the translocation. Our method of sexing according to wing chord length meant that sex was not identified for many individuals and may have biased our sample to exclude smaller males and larger females, potentially limiting our scope of inference. There is reason to believe that in some species males are under increased pressure to migrate faster so they can arrive at breeding areas earlier than females [33], [34]. However, there is less reason to expect faster migration or protandry in the case of less sexually dimorphic species such as the red-eyed vireo (see, [57], [58]. It was not possible to differentiate age in this study but more experienced migrants may be more successful at overcoming the challenges of migration. However, age may not determine dominance for red-eyed vireos [34].

In conclusion, songbird movement decisions during stopover periods are not solely a function of underlying resource distributions but are more complex behavioral responses to a combination of endogenous and exogenous factors. The movement track during each stopover can be thought of as one phase embedded within an organisms' larger lifetime track, the consequences of which will carry over to subsequent phases [1]. For example, if a migrant is obliged to spend more time locating suitable habitat during stopover, she may stay longer than usual to refuel and a penalty may be attached to late arrival at the next stopover site, where resource levels may have been depressed by earlier migrants [12]. Alternatively, if she departs “on time” but with lower fat stores, she will need to stay longer or refuel faster at the next stopover to maintain a “margin of safety” vis-a-vis anticipated energetic demands. If she does not make up lost time, she will arrive late on the breeding grounds potentially jeopardizing opportunities to secure a territory or a mate [3] and if she does not regain reduced fat stores she will arrive in poorer condition on the breeding grounds where she may suffer reduced reproductive success [2].
